# Physician-Reported Benefits and Barriers to Clinical Implementation of Genomic Medicine: A Multi-Site IGNITE-Network Survey

**DOI:** 10.3390/jpm8030024

**Published:** 2018-07-24

**Authors:** Aniwaa Owusu Obeng, Kezhen Fei, Kenneth D. Levy, Amanda R. Elsey, Toni I. Pollin, Andrea H. Ramirez, Kristin W. Weitzel, Carol R. Horowitz

**Affiliations:** 1 The Charles Bronfman Institute for Personalized Medicine and Department of Genetics and Genomic Sciences, The Icahn School of Medicine at Mount Sinai, New York, NY 10029, USA; 2 Pharmacy Department, The Mount Sinai Hospital, New York, NY 10029, USA; 3 Center for Health Equity and Community-Engaged Research, Icahn School of Medicine at Mount Sinai, New York, NY 10029, USA; kezhen.fei@mountsinai.org (K.F.); carol.horowitz@mountsinai.org (C.R.H.); 4 Department of Population Health Science and Policy, Icahn School of Medicine at Mount Sinai, New York, NY 10029, USA; 5 Division of Clinical Pharmacology, Department of Medicine, Indiana University School of Medicine, 950 W. Walnut Street, Indianapolis, IN 46202, USA; kdlevy@iu.edu; 6 Department of Pharmacotherapy and Translational Research, University of Florida College of Pharmacy, Gainesville, FL 32640, USA; aelsey@cop.ufl.edu (A.R.E.); kweitzel@cop.ufl.edu (K.W.W.); 7 Program for Personalized and Genomic Medicine, Departments of Medicine and Epidemiology & Public Health, University of Maryland School of Medicine, Baltimore, MD 21201, USA; tpollin@som.umaryland.edu; 8 Department of Medicine, Vanderbilt University School of Medicine, Nashville, TN 37232, USA; andrea.h.ramirez@vanderbilt.edu

**Keywords:** genetic medicine, pharmacogenetics, chronic disease, genetic testing, physician attitudes, barriers, clinical implementation, clinical utility, physician education

## Abstract

Genetic medicine is one of the key components of personalized medicine, but adoption in clinical practice is still limited. To understand potential barriers and provider attitudes, we surveyed 285 physicians from five Implementing GeNomics In pracTicE (IGNITE) sites about their perceptions as to the clinical utility of genetic data as well as their preparedness to integrate it into practice. These responses were also analyzed in comparison to the type of study occurring at the physicians’ institution (pharmacogenetics versus disease genetics). The majority believed that genetic testing is clinically useful; however, only a third believed that they had obtained adequate training to care for genetically “high-risk” patients. Physicians involved in pharmacogenetics initiatives were more favorable towards genetic testing applications; they found it to be clinically useful and felt more prepared and confident in their abilities to adopt it into their practice in comparison to those participating in disease genetics initiatives. These results suggest that investigators should explore which attributes of clinical pharmacogenetics (such as the use of simplified genetics-guided recommendations) can be implemented to improve attitudes and preparedness to implement disease genetics in care. Most physicians felt unprepared to use genetic information in their practice; accordingly, major steps should be taken to develop effective clinical tools and training strategies for physicians.

## 1. Introduction

Genetic medicine applications in clinical care have been stimulated by an increasing knowledge of the influence of genetic variation on human health as well as the rapid and scalable advances in technologies that interrogate genetic variants [1]. The promise of genomic medicine is to optimize the diagnostic, risk prediction, as well as prevention and treatment decision-making processes in clinical care. There is an ever-expanding ability to diagnose and/or to predict disease using genetic information for both classic “Mendelian” conditions, such as cystic fibrosis, and for common diseases with varying degrees of hereditary genetic etiology, such as cancer, cardiovascular disease, and diabetes [1,2,3,4]. In addition, to date, over 200 (i.e., 10%) of all Food and Drug Administration (FDA)-approved medications contain pharmacogenetic (PGx) information in the label [5]. Many of these drug–gene interactions are clinically “actionable”, meaning they merit inclusion in the clinical decision-making process. The growing number of Clinical Pharmacogenetics Implementation Consortium (CPIC) guidelines [6] and those from other pharmacogenetics guideline-writing groups [7,8,9,10,11,12] is a testament to the expanding robust evidence demonstrating the value of adding genetic information to the clinical decision-making process.

Despite enthusiasm about the potential for genomics to improve patient care, barriers to widespread adoption in clinical practice exist. Scientific, educational, ethical, legal and social issues, information technology, and reimbursement challenges have all been identified as potential barriers preventing healthcare providers from effectively adopting genomic medicine [13]. Genomic medicine is increasingly expanding beyond the purview of genetic counselors and genetic medicine specialists. Not only are there a limited number of these experts [14,15], genetic testing for disease risk and appropriate medication use are increasingly seen as part of general medical care by primary care providers and medical specialists. However, these providers have had limited genetics training and may be unprepared to help their patients decide whether genetic testing is needed, which test(s) to order, how to interpret the results, and apply said results to improve patient care and outcomes [16].

The National Institutes of Health (NIH) funded six projects and a coordinating center to comprise the IGNITE (Implementing GeNomics In pracTicE; www.ignite-genomics.org) Network. The objectives of the network are to develop, explore, and disseminate practice models and tools to facilitate seamless integration of genomic medicine into practice [17]. The IGNITE network covers a wide range of genomic medicine implementation studies in diverse clinical settings. Three of the six sites are conducting clinical pharmacogenetics (PGx) implementation studies. Of the remaining three sites, one is studying extensive family history testing, one chronic kidney disease genetics risk prediction, and one identifies individuals with highly penetrant genetic or monogenic subtypes of diabetes mellitus. The diversity in projects and sites presents an important opportunity to investigate genomic medicine adoption barriers among non-genetic specialists and to specifically compare provider attitudes and barriers among the different types of genetic testing (pharmacogenetics vs. disease-risk prediction or diagnostic refinement) as well as barriers based on provider-specific characteristics (i.e., years of practice experience, gender, and race/ethnicity). This may provide important insight into the types of barriers that must be overcome to ensure the success of genomic medicine in diverse clinical settings. Therefore, we assessed physician attitudes toward genomic medicine adoption across the IGNITE network to identify barriers to genomic medicine adoption among diverse clinical settings.

## 2. Methods

IGNITE’s Common Measures Workgroup developed 14 constructs of effective genomic medicine implementation based on the Consolidated Framework for Implementation Research. This framework guides systematic assessment of multilevel implementation contexts to identify factors that influence intervention implementation and effectiveness [18,19]. We designed survey items based on medical literature [20,21,22,23] using these constructs as well as queries for provider and site characteristics. The survey instrument was tested on a small group of physicians and investigators at the various participating sites and revised accordingly based on feedback from the testers. No further psychometric assessment was conducted. All but one IGNITE site incorporated all or a subset of these survey items into their project-specific provider surveys. Depending on the project design at each site, the survey was either administered in person or electronically between 2014 and 2016. Study investigators recruited physicians for the surveys at regularly scheduled committee or other meetings, grand rounds or other educational presentations, or via emails with a link to the survey, with specific recruitment methods varying among sites. We tailored generic questions to the projects without losing the theme and intent of the question. For example, the question “I am confident in my ability to use the results of a pharmacogenetic test” was revised at the monogenic diabetes site to read “I am confident in my ability to use the results of monogenic diabetes genetic testing” as shown in Table 1.

## 3. Data Analysis

The majority (65%) of the respondents from one chronic disease site were medical residents. However, there were only three medical resident responses from all three pharmacogenetics sites combined. A recent study from the chronic disease site with the majority of resident responses found that medical residents’ opinions on genomic medicine were significantly different from that of attendings, who were excluded from this study. We used descriptive analyses for provider demographics, means, and standard deviations for continuous variables as well as frequencies and proportions for categorical variables. Bivariate analyses of *t*-test or fisher’s exact test and χ² tests for continuous and categorical variables respectively were conducted to compare by project type, clinical setting, gender, years in practice, gender, and ethnicity/race. Multivariate logistic regression models were run to assess significantly different opinions expressed by different providers by adjusting potential demographic confounders. All analyses were done using SAS 9.4 software (SAS Inc., Cary, NC, USA). Statistical significance was set at the 0.05 level.

## 4. Results

### 4.1. Demographics

The 285 physicians from five institutions who completed the survey were from two disease genetics projects (73%) and the three PGx implementation projects (27%). All respondents were directly involved in the care of patients enrolled in the IGNITE-funded project at the various institutions, but few (<1%) of the respondents were study investigators. Of the 285 respondents, 51% practiced in an academic setting and 49% in a community setting. The Institutional Review Board (IRB) at one study site did not allow collection of physician demographics, thus we are missing 47 (61%) of the pharmacogenetics physicians’ gender and race. Of the available demographics, about half (49%) were males, 47% White, 21% Asian, 8% Black, 4% Hispanic Latino, and 19% did not report their race/ethnicity (Table 2).

### 4.2. Site Characteristics

All sites included physicians from outpatient clinics; three had inpatient physicians, and two engaged emergency room physicians. Additionally, four sites recruited physicians in academic settings, and all five sites had responses from community physicians.

### 4.3. Physicians’ Perception of the Clinical Usefulness of Genetic Testing

Two thirds of physicians agreed that genetic or PGx testing is relevant to their clinical practice and one third believed that having access to genetic risk information during patient care would significantly improve their ability to care for their patients (Table 3).

### 4.4. Physician Training and Preparedness to Use Genetic Testing 

Only one third of respondents agreed that their training prepared them to work with “genetically high-risk” patients, and even fewer (15%) felt confident in their ability to use genetic results in practice. 

Physician awareness of resources: Only about one fourth (23%) of physician respondents agreed they could find and use reliable sources of information to understand and communicate genetic risk in the care of their patients. 

### 4.5. Comparison of Pharmacogenetics and Disease Genetic Testing Sites

Physicians from the pharmacogenetics sites were significantly different from those surveyed from the disease genetics sites in regards to their practice settings and their years in practice. The pharmacogenetics sites’ respondents were mostly community practitioners, whereas the majority at the disease genetics sites were academic physicians, *p* < 0.0001 (Table 2). Moreover, the physicians surveyed at the pharmacogenetics sites had mostly (79%) practiced for five or less years in comparison to 69% of physicians from disease genetics sites, who had more than five years of practice experience, *p* < 0.0001 (Table 2). We are unable to comment on differences between the groups in regards to gender or race due to significant missing data. Comparisons of physician opinion by available demographics are presented in Table 2.

Physicians at the PGx sites were more likely than physicians at the disease genetics sites to perceive genetic testing as clinically useful (77% vs. 64%, *p* = 0.04) and to believe that having access to genetic risk information would improve their ability to care for patients (70% vs. 21%, *p* < 0.0001). More physicians participating in PGx practice stated that they felt prepared to incorporate genetics into their practice (58% vs. 22%, *p* < 0.0001), and were more aware of relevant practice resources (32% vs. 19%, *p* = 0.01) in comparison to those physicians focusing on disease genetics (Table 3). Although only 15% of all respondents felt confident in using genetic test results, pharmacogenetics site physicians were more likely to be confident (30% vs. 10%, *p* < 0.0001).

### 4.6. Years of Practice

Physicians with five or less years in practice were more likely than those who had practiced for more than five years to report that their training had prepared them to care for genetically “high-risk” patients (41% vs. 25%, *p* = 0.006). 

Multivariable logistic regression models (Table 4) showed that physicians at PGx sites were more likely (Odds Ratio (OR) = 12.4, 95% Confidence Interval (CI): 5.9–26.0) than physicians from disease genetic sites to agree that having access to genetic testing data would improve their patient care after adjusting for practice setting and years of practice. Although models predicting clinical usefulness and ability to find reliable information were poorly fit, physicians from PGx sites were more likely to agree with these statements than physicians from disease genetic sites. Race and gender were not included in this multivariable logistic regression analysis due to missing data from the majority of the respondents at the PGx sites.

## 5. Discussion

Personalized medicine is rapidly gaining ground, especially in the United States, where multiple government- and privately funded consortia are developing tools, methodologies, and best practices for effective dissemination and implementation across diverse populations and clinical settings [17,24,25,26]. Clinical providers have been identified as a key stakeholder group required to drive the successful adoption of genomic medicine into practice. As such, their perceptions, attitudes, and preparedness to deal with genetic test results in practice should be well characterized, with needs identified and addressed to ensure successful adoption [27].

Studies have repeatedly reported that physicians perceive they lack the knowledge and skillsets to implement genomic medicine in their practices; despite this, physicians consider genetic information to be relevant [28,29,30,31]. This finding was confirmed in the present study. Overall, many of the respondents in this study perceived that their medical training did not prepare them for clinical adoption of genomic medicine, were not confident in using this information, and were unable to locate or effectively use additional resources for genetic tests. Owing to the field of genetics and genomics’ successes, such as the completion of the Human Genome Project among others, it is widely accepted that the incorporation of genetics content into formal medical school curricula is imperative to equip future physicians to provide optimal patient care [32]. As the majority of medical schools have included some basic genetics content in their preclinical courses [33] for decades and providers still feel unprepared, this education does not appear to be sufficient for medical practice in this era of personalized medicine. Studies have consistently demonstrated that practicing physicians perceive that they lack the knowledge or confidence to care for genetically “high-risk” patients [29,30,34]. A subset of medical schools have developed advanced or genomics-focused tracks for their students, and some continue genetics instruction into the clinical years; however, the lack of knowledgeable clinical preceptors may be a challenge for programs adopting this approach [25].

Our study revealed significant differences in physicians’ attitudes and perceptions based upon the type of genetic testing they used clinically (i.e., physicians conducting pharmacogenetic tests versus those using genetic tests to predict disease risk or refine diagnosis). Physicians from sites implementing pharmacogenetic testing had more positive attitudes towards genetic testing, were more prepared and confident in their abilities, and reported a higher awareness of additional resources for genetic testing in comparison to those physicians implementing disease genetics testing. To the best of our knowledge, this is the first multi-site provider attitudes survey which compares physician responses between sites either in the process of clinically implementing or introducing via clinical research pharmacogenetics or disease genetics. Notably, however, the diseases studied (susceptibility to chronic kidney disease and diagnosis for monogenic diabetes) were diverse within this group. Although physicians with fewer practice years have been shown to be more supportive of the clinical utility of genetic medicine [35], and the physicians at the pharmacogenetics sites in the current study are more likely to support the clinical utility of genetic medicine, our multivariate analyses showed that, even after controlling for years in practice, PGx providers retained more positive attitudes (Table 4). Thus, it will be important to identify other factors inherent in clinical implementation of PGx testing, such as the easily accessible, peer-reviewed CPIC guidelines as well as the availability of alternative therapies and sample implementation tools, such as clinical decision support templates available on www.cdskb.org and “how-to” guides developed by the IGNITE network (www.ignite-genomics.org). In all, the study results demonstrate that physicians’ perceptions and readiness for testing are significantly more favorable towards the clinical use of pharmacogenetic testing as compared with genetic testing for chronic disease. 

Study findings support the validity of longstanding predictions that pharmacogenetic testing would be more readily adopted in the clinical environment if certain conditions were met, including, a high proportion of the population is affected, the availability of discrete and actionable examples with robust evidence and clinical guidelines, the established benefits of testing in select populations, the potential for testing to improve patient safety, and the low likelihood of stigmatization and psychological stress associated with pharmacogenetic testing [6,36,37]. Additional clinical resources, such as the inclusion of pharmacogenetic information in FDA labeling for hundreds of medications [38], the creation of the pharmacogenomics knowledgebase (PharmGKB; https://www.pharmgkb.org) [39], and the NIH-funded consortia efforts focused on developing tools and dissemination strategies for pharmacogenetics [17,40,41] have helped to advance this field. A future area for investigation includes the role of clinical decision support provided in influencing the confidence of the provider.

Results of the current study also suggest that recent medical graduates may be more likely to be ready to use and feel comfortable with genetic testing in practice. Physicians with five or less years of medical practice experience favored genetic testing and were more prepared and confident in their abilities to incorporate genetic information in the clinical decision-making process than their senior colleagues. Selkirk et al.’s single academic center survey of physicians on their preparedness for integration of genomic medicine into practice [42] demonstrated that those with prior experience with genetic testing for pharmacogenetics and common disease had a statistically higher mean level of understanding of genetics in comparison with those without prior experience [42]. Nonetheless, the authors did not evaluate the genetics knowledge differences between those with prior experience with disease genetics versus prior pharmacogenetics testing experience. We hypothesize physicians who have been in practice longer are less likely to have received education in chronic disease genomics or pharmacogenetics during their medical school training in comparison with recent graduates. In recent years, genomics education has been increasingly incorporated into medical and other health professional curricula [43,44]. However, the inclusion of genomics content in formal medical training does not always correlate with physicians’ preparedness to adopt genetics in practice, as shown in the Hauser et al. study of physicians treating African-American patients tested for *APOL1* variants to predict their risk of chronic kidney disease [35]. Authors of this study recommended that processes that will facilitate genetic testing, the return of results, clinical decision support tools to aid physicians, as well as additional training may be needed to build physician confidence to adopt genetic testing in practice. On the contrary, previous reports have shown that physicians with more years in practice were more likely to have encountered at least one patient with genetic testing and, accordingly, were more comfortable discussing the benefits and limitations of genetic testing with their patients [23,42,45,46]. In these other studies, recent graduates reported a lack of both knowledge and comfort in implementing genomic medicine [42,45,47]. Several papers postulate that findings such as these may actually be driven by an increased awareness and understanding of knowledge limitations in recent graduates when compared with more senior physicians, which leads to relatively less comfort with integrating genetics into medicine [45,47].

Although our findings on the clinical usefulness of genomic medicine are comparable to other studies [28,30], the results may not be generalizable to all health care providers, such as medical residents, nurse practitioners, pharmacists, and physician assistants, because responses from these providers were excluded from our analysis as a result of low sample sizes. Other limitations of the present study include the low number of common survey items from all five sites which, in turn, limits our insights into the attitudes and perceptions of physicians towards genomic medicine implementation programs and the use of subjective rather than objective assessments to assess knowledge and genomic medicine preparedness. Moreover, clinical utility of *APOL1* testing for chronic kidney disease risk in African ancestry patients may differ from using genetic testing to diagnosis monogenic diabetes and, as such, may limit the interpretation of the results of this study.

## 6. Conclusions

The results of this study, which suggest that physicians in sites offering pharmacogenetic testing were more favorable to clinical implementation of genomic medicine versus those at the chronic disease genetics sites, is novel and warrants further research. Perhaps, the strides and lessons learned from pharmacogenetics clinical advancements in the last decade, such as clinical decision support tools, among others, can be used as a template for other genomic medicine examples to enhance provider uptake and program successes [48,49,50]. Moreover, the clinical course of action needed in the chronic disease genetics examples may not be as clear as that of the pharmacogenetics examples. The former can lead to complex clinical decisions and, to date, genetics-guided clinical guideline for chronic kidney disease does not yet exist. There are guidelines emerging from the International Society for Pediatric and Adolescent Diabetes and the American Diabetes Association for monogenic diabetes [4,51], but applying these guidelines is less straightforward than recommendations from the Clinical Pharmacogenomics Implementation Consortium (CPIC) [52]. The majority of physicians in our sample believe that genetics is a viable clinical tool and yet are unable to tap into this rich resource due to their perceived lack of adequate knowledge, skills, and supporting resources for practicing physicians. It is important to uncover and address barriers vital to the successful clinical adoption of genomic medicine. The implementation of interactive and practice-based educational initiatives targeting practicing physicians, which is supplemented by easily accessible supporting materials and clinical decision support tools, may be the direction that clinical genetics implementation programs need to take to ensure physician buy-in and practice adoption.

## Figures and Tables

**Table 1:** Survey tools administered at the Implementing GeNomics In pracTicE (IGNITE) sites for this study.

	Pharmacogenetics	Disease Genetics	
		Chronic Kidney Disease	Monogenic Diabetes
Clinical Usefulness	I believe that pharmacogenetic testing is relevant to my current clinical practice.	Genetic testing for risk for common diseases offers information that is clinically useful.	I believe that monogenic diabetes genetic testing is relevant to my current clinical practice.
	Pharmacogenetic data will improve my ability to care for patients.	Having access to *APOL1* genetic risk information during patient care will significantly improve my ability to care for patients.	Having access to monogenic diabetes genetic testing will improve my ability to care for patients.
Training and Preparedness	My training has prepared me to treat patients whose family history/genetics place them at high risk for medical conditions.	My training has prepared me to work with patients at high risk for genetic conditions.	My training has prepared me to treat patients whose family history/genetics place them at high risk for monogenic diabetes.
	I am confident in my ability to use the results of a pharmacogenetic test.	I am confident in my ability to use the results of an *APOL1* genetic test.	I am confident in my ability to use the results of monogenic diabetes genetic testing.
Awareness of resources	I can find/use reliable sources of the information I need to apply pharmacogenetics while caring for patients.	I can find/use reliable sources of the information I need to understand and communicate *APOL1* genetic risk while caring for patients.	I can find/use reliable sources of the information I need to apply monogenic diabetes genetic testing while caring for patients.

**Table 2:** Demographics of the survey participants.

	All Respondents	Disease Genetics Sites	Pharmacogenetics Sites	*p*-Value
**Gender**	*n* = 285	*n* = 208	*n* = 77	<0.0001
Male	139 (49%)	126 (61%)	13 (17%)	
Female	92 (32%)	75 (36%)	17 (22%)	
Unreported	54 (19%)	7 (3%)	47 (61%)	
**Practice Setting**				<0.0001
Academic	145 (51%)	121 (58%)	24 (31%)	
Community	140 (49%)	87 (42%)	53 (69%)	
**Race**				<0.0001
White	135 (47%)	125 (60%)	10 (13%)	
Black	24 (8%)	17 (8%)	7 (9%)	
Asian	59 (21%)	48 (23%)	11 (14%)	
Hispanic	12 (4%)	10 (5%)	2 (3%)	
Other/Unreported	55 (19%)	8 (4%)	47 (61%)	
**Years in Practice**				<0.0001
0–5 years	124 (44%)	63 (30%)	61 (79%)	
>5 years	160 (56%)	144 (69%)	16 (21%)	
Unreported	1 (0.4%)	1 (0.5%)	0	

**Table 3:** Physicians attitudes and perceptions towards genomic medicine implementation.

		%	Project Type		Site Type		Years in Practice	
		*n* = 285	*n* = 77	*n* = 208	*n* = 145	*n* = 140	*n* = 124	*n* = 160
		Overall	PGx	Disease Genetics	Academic	Non-Academic	≤5 years	>5 years
Clinical Usefulness	Genetic testing for risk of common diseases offers information that is clinically useful/I believe that PGx testing data is relevant to my current clinical practice.	67	77	64 *	63	71	72	64
	Having access to genetic risk information during patient care will significantly improve my ability to care for patients/ PGx data will improve my ability to care for patients.	35	70	21 ***	32	38	41 **	30
Training and Preparedness	My training has prepared me to work with patients at high risk for genetic conditions.	31	58	22 ***	24 *	38	41 **	25
	I am confident in my ability to use the results of a genetic test.	15	30	10 ***	10 *	19	16	14
Awareness of resources	I can find/use reliable sources of the information I need to understand and communicate genetic risk while caring for patients/PGx genetic risk while caring for patient.	23	32	19 *	23	22	24	21

* *p* = 0.01–0.04; ** *p* = 0.001–0.007; *** *p* < 0.0001.

**Table 4:** Multivariable logistic regression models predicting physician agreement to the following statements.

**Genetic Testing for Risk of Common Diseases Offers Information That Is Clinically Useful/I Believe that PGx Testing Data Is Relevant to My Current Clinical Practice.**			
	**Odds Ratios**	**95% Wald**	
		**Confidence Limits**	
PGx vs. Disease Genetics	1.635	0.841	3.176
Practice setting: Academic vs. Non-academic	0.763	0.447	1.305
Practice: 0–5 years vs. >5 years	1.143	0.638	2.046
Wald = 5.466, df = 3, *p* = 0.1607; c = 0.602			
**Having access to genetic risk information during patient care will significantly improve my ability to care for patients/PGx data will improve my ability to care for patients.**			
	**Odds Ratios**	**95% Wald**	
		**Confidence Limits**	
PGx vs. Disease Genetics	12.42	5.929	26.017
Practice setting: Academic vs. Non-academic	1.06	0.58	1.938
Practice: 0–5 years vs. >5 years	0.526	0.258	1.073
Wald = 51.0872, df = 3, *p* < 0.0001; c = 0.74			
**I can find/use reliable sources of the information I need to understand and communicate genetic risk while caring for patients/PGx genetic risk while caring for patient.**			
	**Odds Ratios**	**95% Wald**	
		**Confidence Limits**	
PGx vs. Disease Genetics	2.461	1.237	4.897
Practice setting: Academic vs. Non-academic	1.243	0.67	2.307
Practice: 0–5 years vs. >5 years	0.854	0.433	1.686
Wald = 7.1101, df = 3, *p* = 0.0685; c = 0.592			
